# FAM46C controls antibody production by the polyadenylation of immunoglobulin mRNAs and inhibits cell migration in multiple myeloma

**DOI:** 10.1111/jcmm.15078

**Published:** 2020-03-06

**Authors:** Ana Belén Herrero, Dalia Quwaider, Luis Antonio Corchete, Maria Victoria Mateos, Ramón García‐Sanz, Norma C. Gutiérrez

**Affiliations:** ^1^ Haematology Department Institute of Biomedical Research of Salamanca (IBSAL) University Hospital of Salamanca Salamanca Spain; ^2^ Cancer Research Center‐IBMCC (USAL‐CSIC) Salamanca Spain; ^3^ Center for Biomedical Research in Network of Cancer (CIBERONC) Salamanca Spain

**Keywords:** FAM46C, immunoglobulin, migration, multiple myeloma, unfolded protein response

## Abstract

*FAM46C*, frequently mutated in multiple myeloma (MM), has recently been shown to encode a non‐canonical poly(A) polymerase (ncPAP). However, its target mRNAs and its role in MM pathogenesis remain mostly unknown. Using CRISPR‐Cas9 technology and gene expression analysis, we found that the inactivation of FAM46C in MM down‐regulates immunoglobulins (Igs) and several mRNAs encoding ER‐resident proteins, including some involved in unfolded protein response and others that affect glycosylation. Interestingly, we show that FAM46C expression is induced during plasma cell (PC) differentiation and that Ig mRNAs encoding heavy and light chains are substrates of the ncPAP, as revealed by poly(A) tail‐length determination assays. The absence of the ncPAP results in Ig mRNA poly(A) tail‐shortening, leading to a reduction in mRNA and protein abundance. On the other hand, loss of FAM46C up‐regulates metastasis‐associated lncRNA MALAT1 and results in a sharp increase in the migration ability. This phenotype depends mainly on the activation of PI3K/Rac1 signalling, which might have significant therapeutic implications. In conclusion, our results identify Ig mRNAs as targets of FAM46C, reveal an important function of this protein during PC maturation to increase antibody production and suggest that its role as a tumour suppressor might be related to the inhibition of myeloma cell migration.

## 1 INTRODUCTION

Multiple myeloma (MM), the second most common haematological malignancy, arises from the abnormal proliferation of immunoglobulin‐secreting clonal malignant plasma cells (PCs).^1^, ^2^ Genomic studies have demonstrated the genetic complexity of this cancer, which is characterized by marked clonal heterogeneity.^3^ Moreover, the role of the bone marrow (BM) microenvironment is crucial for the survival, migration and eventual dissemination of the disease.^4^, ^5^, ^6^, ^7^ Recent progress in identifying somatic mutations using next‐generation sequencing has revealed novel mutations in genes whose function in MM pathogenesis is largely unknown, such as *FAM46C* and *DIS3*.^8^

*FAM46C* is one of the most commonly mutated genes in MM, with somatic point mutations having been identified in about 10% of newly diagnosed MM cases.^9^, ^10^ The vast majority of these mutations are of an inactivating nature, frameshift or non‐sense mutations, which indicates that *FAM46C* may function as a tumour suppressor.^9^ In addition, *FAM46C* is located in cytoband 1p12, which is known to be deleted in approximately 20% of MM patients. Loss of heterozygosity or mutations in *FAM46C* has been associated with shorter survival.^11^ Moreover, the acquisition of *FAM46C* mutations over time, as described in some longitudinal studies, suggests that loss of function of FAM46C might be a progression event in MM.^12^, ^13^

A recent study has demonstrated that *FAM46C* encodes an active non‐canonical poly(A) polymerase^14^ and therefore may increase gene expression by extending the poly(A) tails of some mRNAs in the cytoplasm.^15^ Its authors showed that overexpression of *FAM46C* in human myeloma cell lines (HMCLs) carrying the mutated gene led to polyadenylation and stabilization of several mRNAs, and induced cell death.^14^ Another recent study found that overexpression of *FAM46C* induced substantial cytotoxicity in MM cells, up‐regulated genes involved in unfolded protein response (UPR) and increased Ig light chain production.^16^ However, there are still many functional aspects of the impact of FAM46C loss on MM pathogenesis that remain to be elucidated. Here, we used CRISPR‐Cas9 technology to delete endogenous *FAM46C* in different MM cell lines bearing the wild‐type (WT) gene. The characterization of *FAM46C* KO clones revealed that the loss of FAM46C deregulated some migration‐related factors and sharply increased the migratory ability of MM cells. These findings could explain the relationship between the presence of *FAM46C* mutations/deletions in patients with MM and the progression/poor prognosis of the disease. In addition, we revealed that both immunoglobulin light and heavy chain mRNAs are direct substrates of FAM46C. This finding demonstrates that the loss of polyadenylation activity is the mechanism by which antibody production is decreased in *FAM46C* KO clones.

## 2 MATERIALS AND METHODS

### 2.1 Cell lines

The human myeloma cell lines (HMCLs) JJN3 and RPMI‐8226 were acquired from DMSZ, and U266 from ATCC. The cell lines were cultured as previously described.^17^ Cell line identity was confirmed within the last 3 years by STR analysis with PowerPlex 16 HS System kit (Promega) and online STR matching analysis. The presence of mycoplasma was routinely checked with MycoAlert kit (Lonza), and only, mycoplasma‐free cells were used in the experiments.

### 2.2 CRISPR/Cas9‐mediated generation of *FAM46C* knockout cells

*FAM46C* CRISPR‐Cas9 knockout (KO) plasmids, consisting of a pool of three plasmids, each encoding the Cas9 nuclease and a target‐specific 20 nt guide RNA (gRNA), were obtained from Santa Cruz Biotechnology (sc‐407319). MM cells (1 × 10^6^) were transfected with 5 μg of *FAM46C* CRISPR‐Cas9 KO plasmids, or 5 μg of control CRISPR‐Cas9 Plasmid (sc‐418922), which contained a non‐targeting 20 nt scramble guide gRNA. Transfections were carried out using the Amaxa Cell Line Nucleofector Kit V, the Amaxa Nucleofector device (Lonza), and programs T‐016 for JJN3, X‐005 for U266 and G‐016 for RPMI‐8226. Successful transfection of the CRISPR‐Cas9 plasmids was confirmed by the detection of the plasmid encoded‐green fluorescent protein (GFP). Single GFP + cells were sorted into 96‐well plates 6 days after transfection using a Becton Dickinson FACSCalibur flow cytometer. To favour the growth of single cells, 50% filtered conditioned medium and 20% FBS were added to the culture medium. Isolated clones were expanded in culture over a period of 1 month, in the case of JJN3, or 2 months for U266 and RPMI‐8226, and then genomic DNA was extracted. Clones were analysed by PCR using the primers FAM46C‐FOR and FAM46C‐REV (Table S1). Sanger sequencing was used to evaluate the alterations resulting from non‐homologous end‐joining (NHEJ) at the cut site.

### 2.3 Cell migration and invasion assays

MM cells were washed in serum‐free culture medium and resuspended at a final concentration of 10^6^ cells/mL. In the migration assays, 1.5 mL of cell suspension was seeded in the upper chamber of a 6‐well, 8‐µm pore Transwell plates (Corning‐Costar) and 2.6 mL of RMPI‐1640 with 20% serum was placed in the lower compartment. Invasion assays were performed using BioCoat Matrigel Invasion 24‐well, 8.0‐μm pore Transwell chambers (Corning‐Costar). For these experiments, 500 µL of cells resuspended at 10^6^ cells/mL in serum‐free medium was seeded in the upper chamber, and 750 µL of medium containing 20% serum was used as the chemoattractant in the lower chamber. After 20 hours, cells migrating into the lower chambers were collected, resuspended in 400 μL PBS and counted using a BD Accuri C6 flow cytometer.

### 2.4 RNA extraction and microarray data analysis

Total RNA was extracted from three independent *FAM46C* WT or KO clones using an RNeasy mini kit (Qiagen). RNAs were then processed and used to hybridize Affymetrix PrimeView Human Gene Expression Arrays following the manufacturer's instructions. Raw data were background‐adjusted, normalized and log_2_‐transformed using the RMA algorithm^18^ available in the Affymetrix expression console (v.1.4.1). Microarray data were deposited in Gene Expression Omnibus (GEO) under accession number GSE114984. We compared the gene expression of the three *FAM46C* KO clones with each of the three control samples, resulting in nine comparisons. Genes with an absolute value of the fold change (FC) greater than 1.5 were selected for further analysis. A second approach using an absolute FC > 1.2 was carried out to assess the overrepresentation of gene ontology categories.

### 2.5 Quantitative real‐time polymerase chain reaction (qRT‐PCR) analysis

Total RNA was reverse‐transcribed to cDNA using a cDNA Reverse Transcription Kit from Applied Biosystems. qRT‐PCR was performed using an iQ™ SYBR^®^ Green Supermix kit (Bio‐Rad), the iQ5 PCR detection system and the following gene‐specific primers: FAM46C‐FOR and FAM46C‐REV2, *GAPDH‐FOR* and *GAPDH‐REV* (Table S1)*.* Alternatively, and for other genes, expression was assessed using TaqMan qRT‐PCR assays (Applied Biosystems). Relative gene expression was calculated by the 2^−ΔCt^ method using GAPDH as the reference gene for normalization or 18S rRNA when indicated.

### 2.6 siRNA

HMCLs were transfected with 25 nmol/L of on‐TARGET plus™ control pool or on‐TARGET plus SMART pool Human *FAM46C* (Dharmacon). Transfections were carried out using the Amaxa Cell Line Nucleofector Kit V, the Amaxa Nucleofector device (Lonza) and program G‐016.

### 2.7 Cell proliferation and cell viability assays

Cell proliferation and cell viability in the absence or presence of drugs was assessed by the MTT assay. Apoptosis was measured using annexin V‐fluorescein isothiocyanate/propidium iodide (PI) double‐staining (Immunostep) according to the manufacturer's procedure.

### 2.8 Poly (A) tail‐length determination

mRNA poly(A) tail length was analysed using the USB^®^ Poly(A) Tail‐length assay kit from Affymetrix. Gene‐specific forward primers were as follows: IGKC, SSR4, IGLC, BIP, IGHA1ns, IGHA1s, IGHEns and IGHEs (Table S1).

## 3 RESULTS

### 3.1 Knockout of *FAM46C* slightly affects the gene expression profile

**Figure 1:**
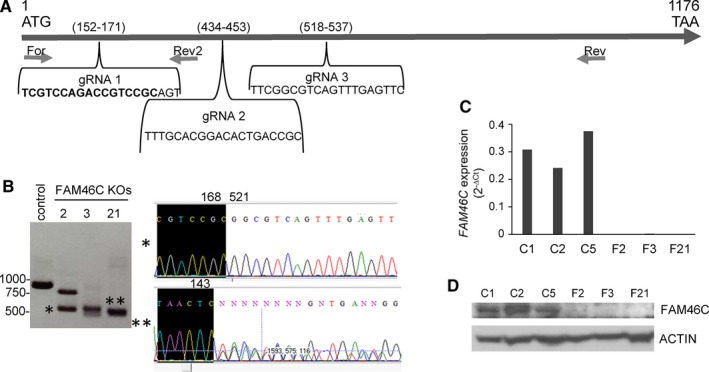
Generation of FAM46C KO clones in JJN3 using CRISPR‐Cas9 technology. A, Schematic representation of *FAM46C* encoding sequence located in exon 2. The positions of gRNAs and oligonucleotides used in the PCRs are shown. B, Analysis of genome editing by PCR and Sanger sequencing. DNA fragments, generated by PCR using genomic DNA, For and Rev primers, were run in an agarose gel (left), purified and Sanger‐sequenced (right). The control band corresponding to the *FAM46C* DNA derived from WT cells. Clones 2, 3 and 21 presented *FAM46C* deletions that resulted in smaller PCR bands; the lower DNA band in KO2 (*) resulted from a deletion that fused nucleotides 168‐521. The upper band was non‐specific, indicating that a homozygous deletion occurred in KO2. Clone KO21 conserved the WT sequence until position 143, and then, different deletions occurred in the two FAM46C alleles. C, qRT‐PCR analysis of *FAM46C* using For and Rev 2 oligos. No *FAM46C* expression was detected in clones F2, F3 and F21 (C indicates control WT clones). D, Western blot of FAM46C protein in WT and KO clones

To investigate the consequences of FAM46C inactivation for the pathogenesis of MM, we employed CRISPR‐Cas9 technology to delete endogenous *FAM46C* in MM cells. The experiments were performed in the JJN3 cell line, which bears a wild‐type (WT) *FAM46C* gene.^10^, ^16^ JJN3 cells were transfected with a pool of three plasmids each encoding a *FAM46C*‐specific gRNA (Figure 1A) or with a control NT gRNA plasmid, and single GFP+ transfected cells were isolated by flow cytometry. The resulting clones were screened by PCR‐Sanger sequencing (Figure 1B), qRT‐PCR (Figure 1C) and Western blot (Figure 1D). Three clones knockout (KO) for *FAM46C* and three control WT clones were selected and used for further studies.

Recent studies have shown that *FAM46C* encodes a non‐canonical mRNA poly(A) polymerase that may affect the stability of its target mRNAs.^14^, ^19^ In order to identify putative FAM46C substrates, total RNAs were extracted from *FAM46C* WT and KO clones and subsequently processed for microarray hybridization. Surprisingly, we found few genes whose levels of expression in all *FAM46C* KO clones changed relative to WT clones. Probe sets corresponding to eight genes (*MAGED1, LRPAP1, RHOBTB1, GPX7, CKAP4, PCOLCE2, CD55* and *HACD1*) were underexpressed in the nine comparisons (three *FAM46C* KO cells compared with three controls) using a FC < −1.5 (Table S2), and 80 probe sets corresponding to 54 genes decreased their expression 1.2‐fold (Table S3). On the other hand, no gene was up‐regulated using a FC > 1.5, although 12 genes were overexpressed 1.2‐fold in all *FAM46C* KO cells compared with controls (Table S4). When we considered at least six comparisons, nine genes were up‐regulated 1.5‐fold: *EIF4E3, SKAP2, CCDC84, PAGE5, PROK2, SORBS2, MALAT1, PLSCR1* and *TRA2* (Table S5).

### 3.2 Inactivation of FAM46C up‐regulates oncogenic lncRNA *MALAT1* and promotes cell migration and invasion in MM

**Figure 2:**
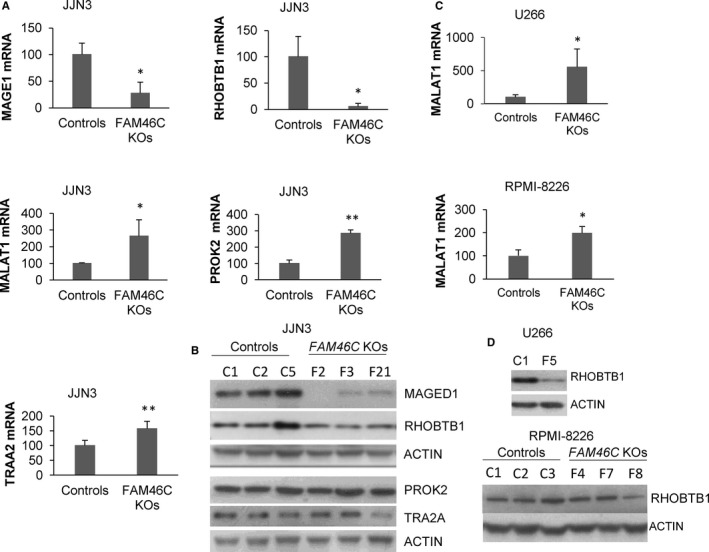
Inactivation of *FAM46C* in JJN3 deregulates the expression of several genes involved in cell migration and invasion. A, mRNA levels of the indicated genes determined by qRT‐PCR. The average expression in the three control clones was taken as 100%, and the average values in the three *FAM46C* KO clones were normalized accordingly. Error bars correspond to the SD (*P ˂ .05, **P ˂ .01 compared with controls). B, Levels of the indicated proteins detected by Western blot. C, Levels of lncRNA MALAT1 in U266 and RPMI‐8226. D, Levels of RHOBTB1 in U266 determined by Western blot

Examining the functions of the genes deregulated in *FAM46C* KO clones, we found that some had previously been associated with cell migration and invasion: *MAGED1* and *RHOBTB1* inhibiting,^20^, ^21^, ^22^ and lnc RNA *MALAT1, PROK2* and *TRA2* promoting these activities.^23^, ^24^, ^25^ The deregulation of these five genes observed in the microarray analysis was validated by qRT‐PCR (Figure 2A). Moreover, reduced levels of MAGED1 and RHOBTB1 proteins were confirmed by Western blot (Figure 2B). Then, we used the CRISPR‐Cas9 technology to delete endogenous *FAM46C* in U266 and RPMI‐8226, which also express wild‐type *FAM46C*. In the same way as in JJN3, three WT and three *FAM46C* KO clones, confirmed by PCR, Sanger sequencing (data not shown) and Western blot (Figure S1), were selected and used in the experiments. Nevertheless, for some reason, U266 *FAM46C* KO clones stopped growing after several passages. RNA was obtained from the three U266 KO clones, but protein extracts only from clone F5. LncRNA MALAT1 was significantly up‐regulated in *FAM46C* KO clones compared with WT clones in both U266 and RPMI‐8226 (Figure 2C), whereas no statistically significant differences were detected in *MAGED1, PROK2* and *TRA2* expression between WT and KO clones (data not shown). An evident down‐regulation of the tumour suppressor protein RHOBTB1 was observed in U266 *FAM46C* KO cells (Figure 2D), as previously found in JJN3 (Figure 2B). However, this effect was not detected in RPMI‐8226 *FAM46C* KO clones.

**Figure 3:**
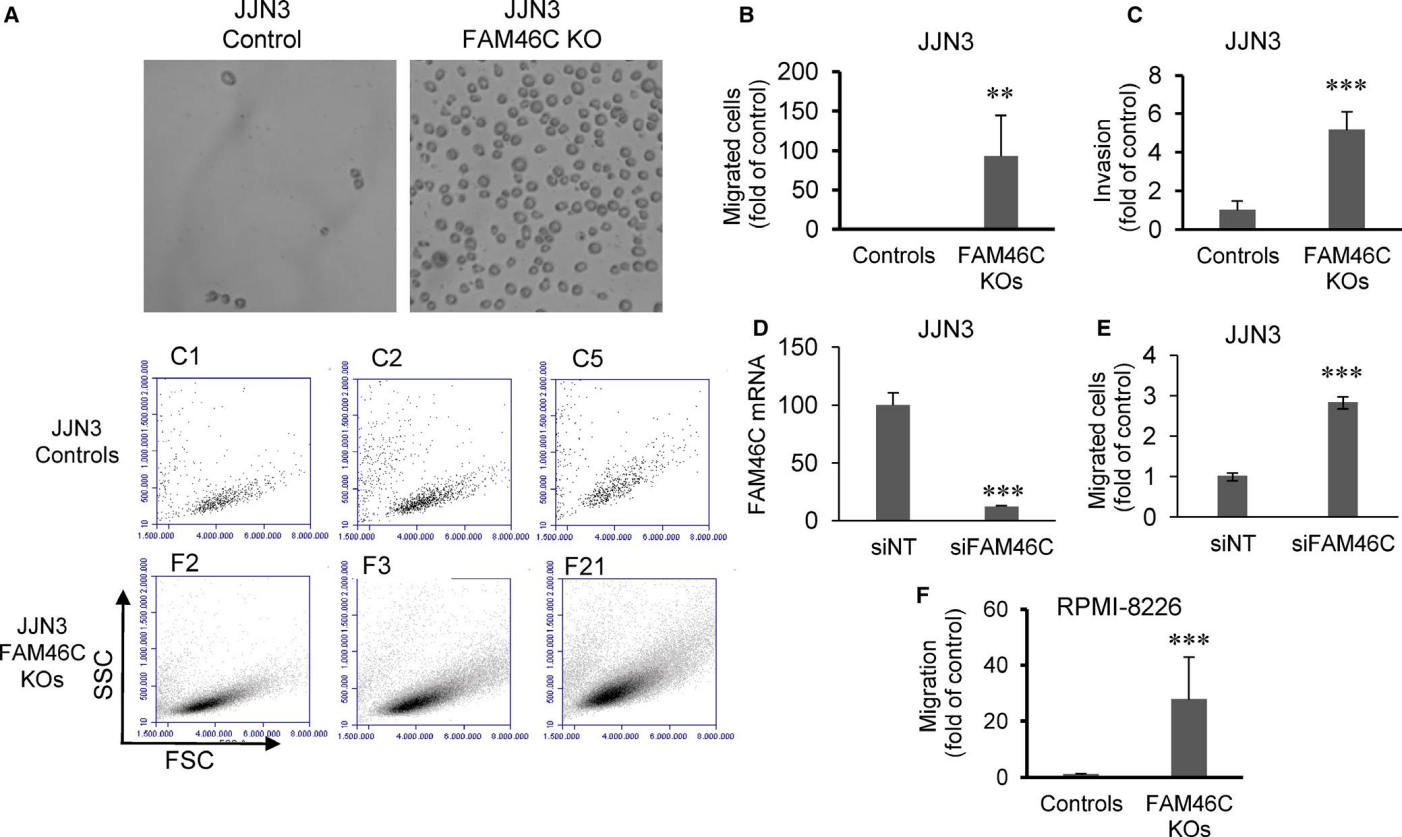
Loss of *FAM46C* promotes cell migration and invasion of MM cells. A, *FAM46C* WT (control) and KO JJN3 cells that migrated from the upper to the lower chamber of the Transwell plates. Cells were photographed (upper panel), centrifuged and counted by flow cytometry (lower panel). B, Cell migration in controls and *FAM46C* KOs cells. The average number of migrated cells in the three control clones was taken as 1, and the values of the three *FAM46C* KO clones were normalized accordingly. C, Cell invasion of JJN3 clones. D, Silencing of *FAM46C* expression in JJN3 by siRNA. mRNA levels were determined by qRT‐PCR. E, Cell migration in JJN3 transfected with non‐targeting siRNA (siNT) or FAM46C siRNA (siFAM46C) assayed 48 h after transfection. F, Cell migration in RPMI‐8226 controls and FAM46C KO clones. All results are presented as the means and SDs of at least three independent experiments (*P ˂ .05, **P ˂ .01, ***P ˂ .001 compared with the correspondent controls)

Deregulation of migration‐related factors in *FAM46C* KO cells prompted us to carry out in vitro cell migration and invasion assays. Interestingly, we found that the number of migratory cells in JJN3 was almost 100 times greater in *FAM46C* KO clones than in WT controls (Figure 3A,B). Moreover, *FAM46C* KO cells also exhibited a greater invasive ability relative to WT cells (Figure 3C). To verify that the increase in cell mobility was independent of putative CRISPR‐Cas9 off‐target effects, we knocked down *FAM46C* expression by siRNA (Figure 3D) and performed cell migration assays. Down‐regulation of *FAM46C* in JJN3 significantly increased the migratory ability of the cells (Figure 3E), confirming that loss of *FAM46C* promotes cell migration. Next, the in vitro cell migration assays were performed in the three RPMI‐8226 *FAM46C* WT and KO clones. We found that the inactivation of the ncPAP induced a significant increase in migration, as previously demonstrated in JJN3 (Figure 3F).

### 3.3 Increased migration of *FAM46C* KO cells depends on PI3K activation

**Figure 4:**
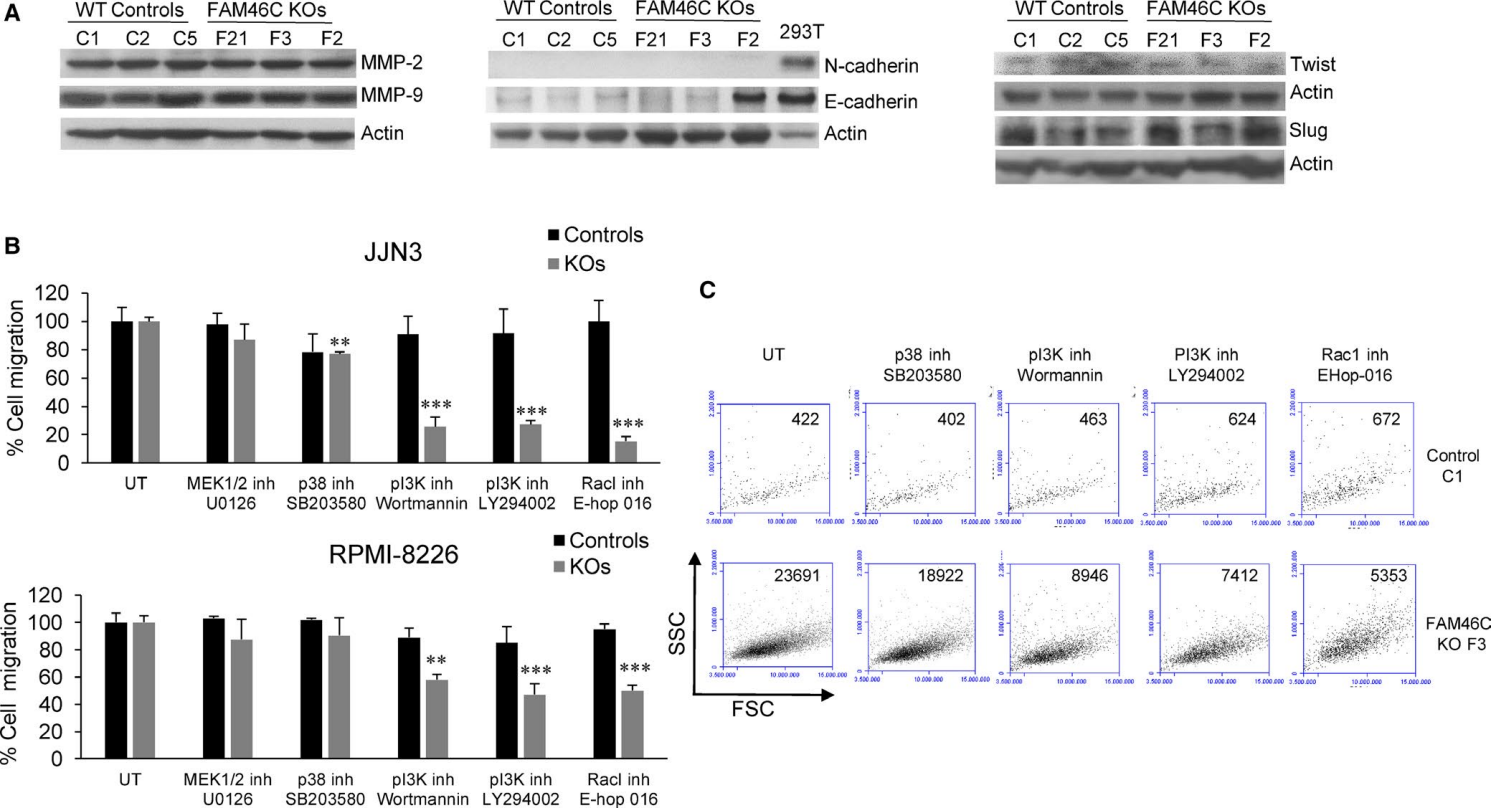
Increased migration of *FAM46C* KO cells is independent of EMT but depends on PI3K‐Rac1 activation. A, Western blot of the indicated proteins in JJN3 *FAM46C* WT and KO clones. Extracts from the HEK293T cell line, known to express both N‐ and E‐cadherins, were used as positive controls. B, Cell migration of *FAM46C* KO cells in the absence (untreated, UT) or in the presence of 10 µmol/L U0126, 1 µmol/L SB203580, 1 µmol/L Wortmannin, 7.5 µmol/L LY294002 or 3 µmol/L EHop‐016. Cells were preincubated for 1 h with the indicated inhibitors and then placed into the upper chamber of the Transwell plates. The number of migrated cells in the UT condition was taken as 100%, and cell migration in the presence of the different inhibitors was normalized accordingly. Error bars correspond to the SD of three independent experiments (**P ˂ .01, ***P ˂ .001). C, Representative dot‐plots showing the JJN3 cells that migrated from the upper to the lower chamber of the Transwell plate

Next, we investigated molecular mechanisms that might underlie the increased migration and invasion of *FAM46C* KO cells. As matrix metalloproteinases (MMPs) are key mediators in cell invasion, we investigated whether the loss of *FAM46C* affected MMPs expression in MM cells. However, similar levels of MMP2 and MMP9 were found in *FAM46C* WT and KO cells (Figure 4A). In MM, an activation of the epithelial‐mesenchymal transition (EMT) similar to the phenomenon observed in solid tumours, which is considered a key process for metastasis, has been described.^26^ EMT is characterized by the loss of E‐cadherin, mediated by the up‐regulation of its repressors such as Slug or Twist, and an increase in N‐cadherin.^27^ Although MM cells are not epithelial cells, some MM cell lines express N and/or E‐cadherin.^28^ We found that JJN3 cells, either *FAM46C* WT or KO, had no detectable level of N‐cadherin, and a very low level of E‐cadherin expression was observed in the different clones, with the exception of clone F2 (Figure 4A). Twist and Slug protein levels did not increase in *FAM46C* KO clones compared with WT cells. These results indicate that the increased rate of migration observed in *FAM46C* KO MM cells seems to be EMT‐independent.

Previous studies have shown that MAPKs and PI3K can also regulate cell migration processes,^29^, ^30^ so we evaluated the influence of different kinase inhibitors on the migration ability of *FAM46C* WT and KO cells. We found that inhibition of MEK1/2 with U0126 did not affect migration of the cells and a small decrease in cell mobility was observed after inhibition of p‐38 with SB203580 in JJN3 *FAM46C* KOs. However, a sharp reduction in cell migration occurred in *FAM46C* KO cells when PI3K was inhibited with Wortmannin or with the specific inhibitor LY294002 (Figure 4B). PI3K acts via diverse downstream signalling components, including the GTPase Rac1 and the kinase Akt (PKB), to promote cell mobility.^31^ We found that migration of *FAM46C* KO cells depended on Rac1 activation in these cells, as revealed by the fact that treatment with the specific inhibitor EHoP‐016 also reduced cell mobility (Figure 4B). Representative dot‐plots of JJN3 WT and *FAM46C* KO cells that migrated from the upper to the lower chamber in the absence or presence of the different inhibitors are shown in Figure 4C. The decrease in cell migration induced by the aforementioned inhibitors was not associated with any effect on cell proliferation, as WT and KO cells did not proliferate in the conditions assayed, neither on cell survival, as apoptosis was not observed at the doses employed (Figure S2).

### 3.4 Knockout of *FAM46C* does not increase proliferation rates or resistance to antimyeloma drugs

**Figure 5:**
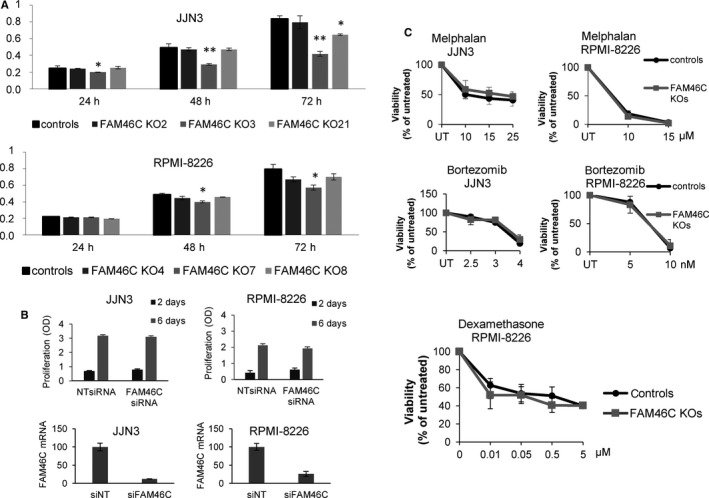
Knockout of *FAM46C* does not increase proliferation rates or affect sensitivity to antimyeloma drugs or the abundance of survival factors. A, Proliferation of control and *FAM46C* KO clones after 24‐, 48‐ and 72‐h growth. B, Proliferation of cells transfected with NTsiRNA or FAM46C siRNA 2 and 6 d after transfection. Cell growth was determined by the MTT assay (OD = optical density). Efficiency of silencing is shown in the lower panel. Error bars correspond to the SD of three independent experiments (**P ˂ .01 compared with controls). C, Viability of the different clones measured by the MTT assay 72 h after treatment with the indicated drugs

To determine whether loss of *FAM46C* affected cell growth rate, JJN3 and RPMI‐8226 clones were cultured and proliferation was tested by the MTT assay. Growth rates did not increase in any of the *FAM46C* KO clones compared with controls. In fact, some of them, but not all, exhibited a slight decrease in growth ability (Figure 5A), suggesting that differences in growth rates may be clone‐dependent and not FAM46C‐dependent. On the other hand, silencing *FAM46C* in JJN3 and RPMI‐8226 did not affect cell growth under the conditions tested (Figure 5B). We then evaluated the effect of common antimyeloma drugs in the different clones, and no significant differences were found in the sensitivity of *FAM46C* WT and KO clones to melphalan, bortezomib or dexamethasone (Figure 5C).

It has recently been reported that depletion of *FAM46C* in the XG1 MM cell line results in increased expression of IRF4, Bcl2 and ERK signalling activation.^16^ The authors proposed that these changes might explain the increased survival to dexamethasone and lenalidomide observed in this cell line. However, we observed similar amounts of these proteins in JJN3, U266 and RPMI‐8226 cell lines in the presence or absence of *FAM46C* (Figure S3), indicating that the survival phenotypes could be cell line‐dependent.

### 3.5 *FAM46C* is up‐regulated during plasma cell differentiation and directly controls Ig production

**Figure 6:**
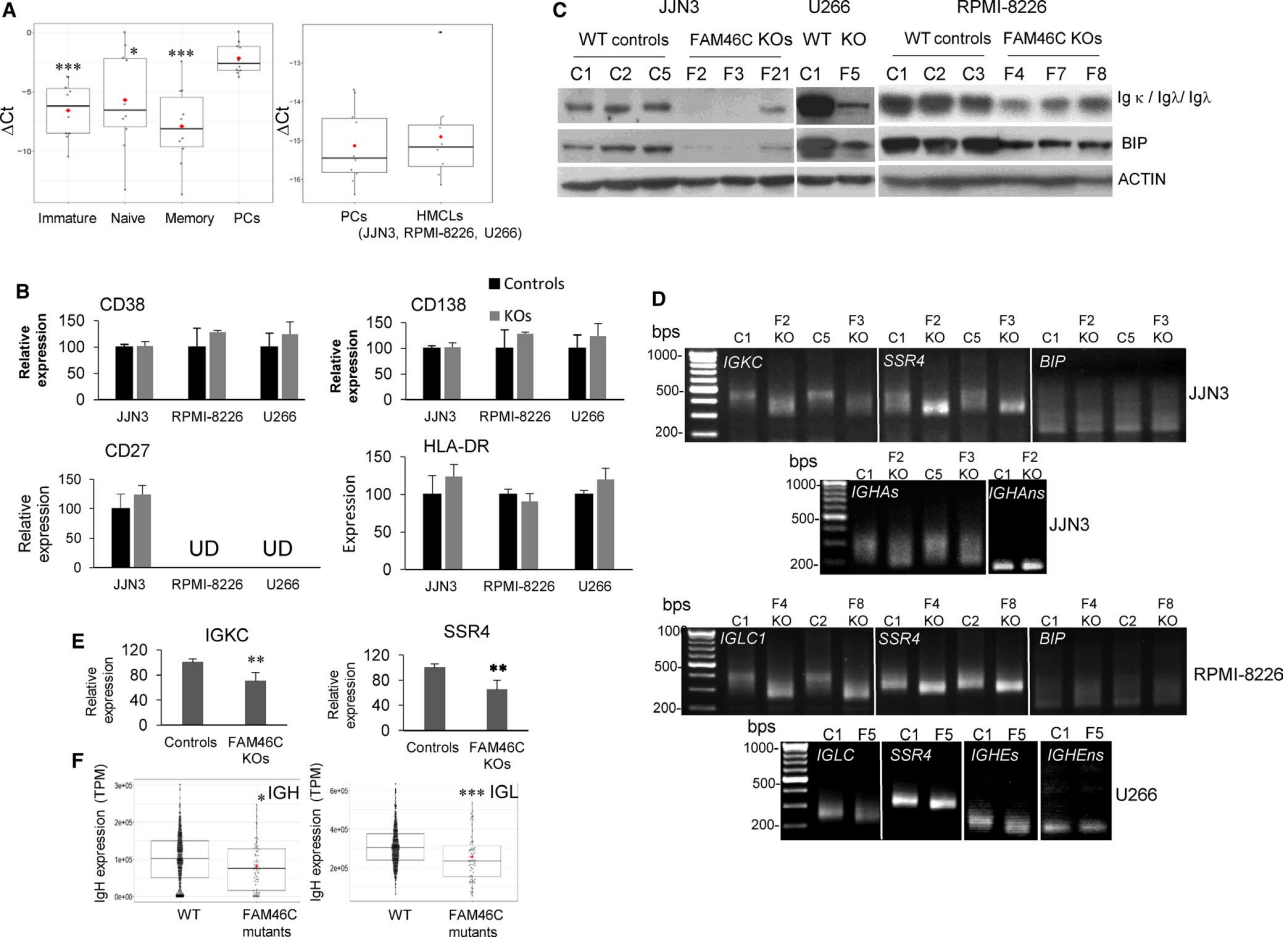
*FAM46C* is up‐regulated during plasma cell differentiation and directly controls Ig production. A, *FAM46C* expression detected by qRT‐PCR in immature B cells, naive B cells, memory B cells and NPC obtained from 10 healthy donors (‐median, ♦ mean); GAPDH was used as the reference gene (left), and 18S rRNA was used for normalization in NPCs and HMCls (right). B, Expression of the indicated genes in JJN3 measured by qRT‐PCR. Average expression in the three control clones was taken as 100%, and the average values of the three *FAM46C* KO clones were normalized accordingly. C, Levels of Ig light chain and BIP in the indicated cell lines detected by blot. D, Determination of polyA tail length of the indicated mRNAs in JJN3, RPMI‐8228 and U266. RNA samples were processed as described in the materials and methods and run in a 2.5% agarose gel (IGHAs and IGHEs indicate secreted mRNAs, IGHAns and IGHEns non‐secreted mRNAs, respectively). E, Relative expression of *SSR4* and *IGKC* in JJN3 WT cells (controls) and *FAM46C* KO cells (*P ˂ .05, **P ˂ .01, ***P ˂ .001). F, Expression of the constant regions of Ig heavy (IGH) and Ig light (IGL) genes in MM patients (WT, n = 559, FAM46C mutants, n = 63) expressed as trimmed means of *M*‐values of normalized counts (*P ˂ .05, ***P ˂ .001, Mann‐Whitney *U* test)

Gene expression profiling of lymphomas from the Emu‐myc transgenic mice identified *FAM46C* among the genes included in the B lymphocyte developmental signature^32^ using the GSEA platform^33^, ^34^ (MSigDB M1487 geneset, Table S6). These results suggested that FAM46C might be involved in PC differentiation, so we first quantified *FAM46C* mRNA levels by qRT‐PCR in four BC populations, immature, naïve, memory B cells and PCs isolated from BM samples obtained from healthy donors.^35^ *FAM46C* expression was significantly higher in PCs than in the earlier stages of differentiation (Figure 6A, left panel) and was similar in PCs and in HMCLs (Figure 6A, right panel). However, when the expression of key genes involved in B‐cell maturation was analysed in *FAM46C* WT and KO cells by qRT‐PCR, no significant differences in their expression were observed between the samples (Figure 6B). These results indicate that loss of *FAM46C* does not revert the state of PC differentiation.

Zhu et al^16^ have recently described that FAM46C affects Ig light chain production. Consistent with this observation, we found in all *FAM46C* KO cells a clear reduction of Ig light chain and BIP protein, which is involved in the correct folding of the proteins in the ER^36^ (Figure 6C). Therefore, we wondered whether Ig and BIP mRNAs might be direct targets of the non‐canonical poly(A)‐polymerase activity of *FAM46C*. As shown in Figure 6D, poly(A) tails of mRNAs encoding Ig Kappa and Ig Lambda constant regions (*IGKC,* in JJN3 and *IGLC1,* in RPMI‐8226 and U266) were clearly shorter in *FAM46C* KO cells than in the corresponding WT controls, revealing that Ig light chain mRNAs are direct substrates of the non‐canonical poly(A) polymerase. In the case of heavy chains, there are two mRNAs, secretory and non‐secretory, which are mainly expressed in PCs and B cells, respectively.^37^, ^38^ We found that IgH secretory mRNAs (*IGHA*1 in JJN3 and *IGHE* in U266) were also targets of FAM46C, but not the non‐secretory IgH mRNAs, since in this case a band of similar size was amplified in both WT and *FAM46C* KO cells (RPMI‐8226 only express Ig light chains). Conversely, we found that poly(A) tail length of BIP mRNA was similar in the different samples. SSR4, a known target of FAM46C,^14^ was used as a positive control. As expected, qRT‐PCR showed that both *SSR4* and *IG* mRNA were less abundant in *FAM46C* KO cells than in the WT clones (Figure 6E). The requirement of FAM46C for Ig production could therefore explain the increased expression of this ncPAP in PCs compared with previous stages of differentiation.

To validate the contribution of FAM46C to Ig production in the clinical setting, we used the RNAseq data corresponding to the MMRF CoMMpass trial (NCT01454297). We compared the levels of monoclonal Ig expression in patients carrying *FAM46C* non‐sense mutations versus those expressing the *FAM46C* WT gene. A lower level of expression of monoclonal Ig, both Ig heavy and light chains, was observed in the patients with mutations of *FAM46C* (Figure 6F).

### 3.6 Inactivation of *FAM46C* decreases ER stress and down‐regulates some genes involved in glycosylation

Functional enrichment analysis of the 54 genes down‐regulated in JJN3 *FAM46C* KO cells (Table S3) failed to identify functional categories. However, the GO‐term cellular component analysis showed that 17 genes encoded proteins located in the ER: *AGA*, *DDOST*, *PDIA6, TMED10, CD55, ERP44, LMAN1, ERGIC3, MYDGF, GPX7, LRPAP1, TXNDC12, FKBP7, EDEM2, CANX, RPN1* and *SSR4* (the known *FAM46C* mRNA substrate). Interestingly, four of them are involved in glycosylation: *DDOST* and *RPN1*, which both encode components of the N‐oligosaccharyl transferase complex, *LMAN1*, involved in glycoprotein transport, *AGA1*, an aspartylglucosaminidase and *SSR4*, involved in translocating proteins across the ER membrane whose mutation causes a congenital disorder of glycosylation (http://www.genecards.org). Five of the genes, *PDIA6, ERP44, TXNDC12, EDEM2* and *CANX,* have a role in tackling ER stress^36^ (http://www.genecards.org). These findings, together with the reduction in the level of BIP protein (Figure 6C), prompted us to consider the hypothesis that the decrease in Ig production, and probably of other proteins, in *FAM46C* KO cells could reduce the secretory cargo of the ER, leading to a diminished level of UPR. To test this, we monitored UPR activation in *FAM46C* WT and KO clones after treatment with the ER stress‐inducer tunicamycin by means of RT‐PCR analysis of spliced/unspliced *XBP1 mRNA*. We observed the appearance of spliced *XBP1* 4 hours after treatment with tunicamycin in all the tested clones, and its subsequent disappearance over time (Figure S4), as previously described.^39^ However, the *XBP1* unspliced form remained more intense at 4 and 8 hours after treatment with tunicamycin in all JJN3 *FAM46C* KO clones compared with WT cells (Figure S4A). In RPMI‐8226, the *XBP1* unspliced forms reappeared after 30 hours of treatment and were also more intense in the *FAM46C* KO than in WT controls (Figure S4B). These results suggest a reduced level of ER stress in *FAM46C* KO cells.

Finally, we took advantage of the MMRF CoMMpass study (research.themmrf.org) and obtained a list of genes down‐regulated in PCs from patients exhibiting *FAM46C* non‐sense mutations. A total of 1681 genes were identified with an FDR < 0.05, of which 909 exhibited an FC < −2 (Table S7). The enrichment analysis using *WebGestalt* identified 13 functional categories (Table S8), and one of them was the ‘response to ER stress’ category, which included 33 genes. On the other hand, cellular component analysis found that 124 genes were located in the ER. When we cross‐checked this list with that including the genes down‐regulated in JJN3 *FAM46C* KO cells (Table S3), 11 genes emerged as being commonly down‐regulated in all *FAM46C* KO clones and in MM patients carrying *FAM46C* mutations (Table S9). Interestingly, three of them participate in UPR (HSPA13, EDEM2 and PDIA6) and three are involved in glycosylation (DDOST, AGA and SSR4). These results suggest that inactivation of this non‐canonical poly(A) polymerase may affect the glycosylation patterns of PCs.

## 4 DISCUSSION

**Figure 7:**
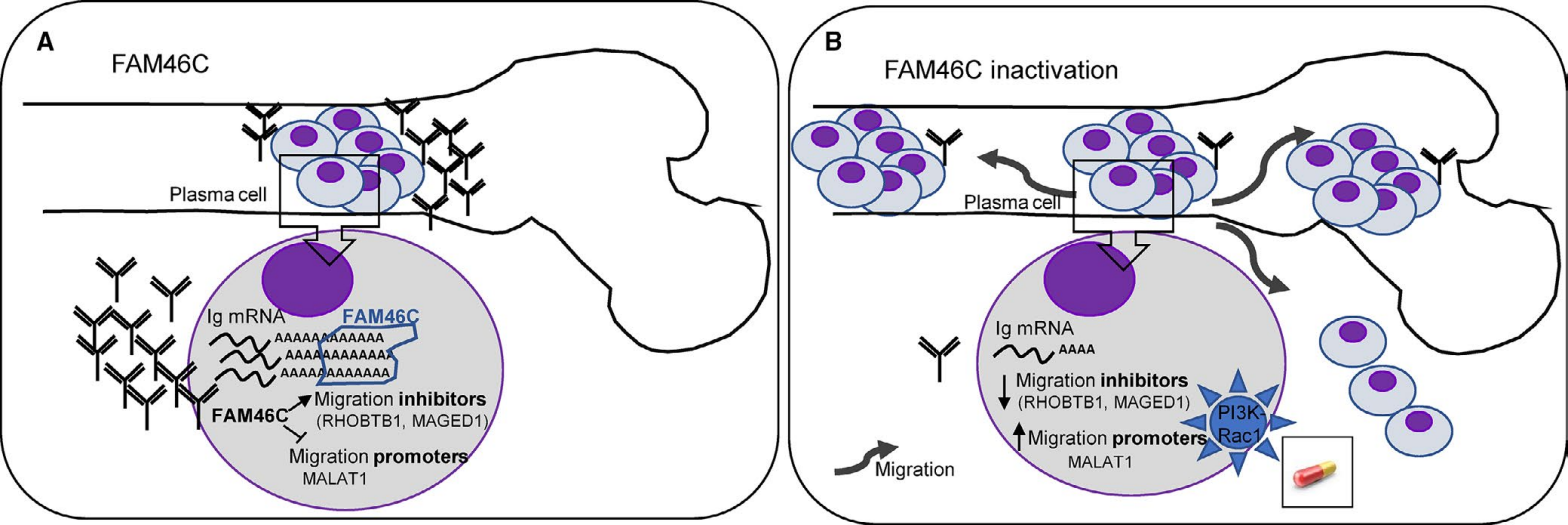
Schematic representation of the functional consequences of *FAM46C* inactivation. A, FAM46C is up‐regulated during PC differentiation to increase antibody production by extending the poly(A) tail of Ig mRNAs. B, Inactivation of the ncPAP reduced Ig poly(A) tail length, the amount of Ig mRNAs and, consequently, the Ig protein levels. Moreover, the lack of FAM46C increases the migratory ability of MM cells, which might explain the role of this gene as a tumour suppressor. The increased rate of migration after *FAM46C* knockout depended on PI3K‐Rac1 activation. Therefore, patients with FAM46C mutations could benefit from PI3K or Rac1 inhibitors

The poly(A) tail plays an important role in the post‐transcriptional control of gene expression as it regulates mRNA transport, stability and translation. In addition to canonical nuclear poly(A) polymerase (PAP), which adds poly(A) tails to most eukaryotic mRNAs, seven non‐canonical PAPs (ncPAPs) have been identified.^15^ *FAM46C*, which is frequently mutated in MM, has recently been described as a new ncPAP.^14^, ^19^ However, its function in myeloma cells remains to be elucidated. In this study, we showed that *FAM46C* is up‐regulated during PC differentiation to increase antibody production by extending the poly(A) tail of Ig mRNAs. Moreover, we demonstrated that inactivation of FAM46C in MM sharply increased the migratory ability of PCs, which might explain the poor prognosis of MM patients with *FAM46C* abnormalities and the role of this gene as a tumour suppressor (Figure 7).

The terminal differentiation of B cells, which bear surface Ig, into antibody‐secreting plasma cells is accompanied by a substantial increase in the abundance of the mRNAs of both Ig heavy and light chains.^40^ It has been reported that this increase is not due to a higher transcription rate but to an extended half‐life of Ig mRNAs.^40^ In the case of IgH chains, two different mRNAs from a single primary transcript exist: B cells produce more mRNA encoding the membrane‐associated protein, while plasma cells contain greater amounts of the Ig mRNA encoding the secreted protein. Regulation of this process requires competing splice and cleavage‐polyadenylation reactions with balanced efficiencies.^37^, ^38^ In this study, we found that poly(A) tails of IgH secretory‐specific mRNAs and Ig light chain mRNAs are controlled by FAM46C. Inactivation of the ncPAP reduced Ig poly(A) tail length and reduced the amount of Ig mRNAs and, consequently, the Ig protein levels. These results indicate that one of the functions of FAM46C in myeloma cells is to increase Ig production, which could explain the up‐regulation of this ncPAP in PCs relative to that observed in earlier stages of B‐cell differentiation. Importantly, a correlation between the presence of *FAM46C* mutations and significantly lower Ig mRNA levels was also observed in MM patients.

GEP of *FAM46C* WT and KO clones revealed that few genes were deregulated after *FAM46C* inactivation. These results indicate either that loss of *FAM46C* slightly affects mRNA stability under normal culture conditions and do not significantly alter RNA steady‐state levels, or that FAM46C has few RNA targets. Our results contrast with those of Mroczek et al,^14^ who found 538 genes commonly up‐regulated by FAM46C overexpression in two HMCLs carrying mutations in *FAM46C*. They also found hundreds of mRNAs that shifted towards longer poly(A) fractions in response to forced FAM46C expression. The most likely explanation for these apparently contradictory results is that ectopic overexpression of FAM46C may result in forced poly(A) addition to mRNAs. Another difference concerns the increased cell proliferation after *FAM46C* silencing previously reported,^14^, ^16^ which was not observed in our study, neither by silencing nor by complete inactivation. These discrepancies might depend on cell lines, clones or experimental conditions. In any case, the top hit FAM46C substrate described by Mroczek et al, SSR4, was also identified in our study, and several ER‐resident proteins were found to be affected by FAM46C in both studies. SSR4 was down‐regulated secondarily to the shortening of its mRNA poly(A) tail after *FAM46C* inactivation, which confirms this mRNA as a *bona fide* FAM46C substrate. It is of note that *SSR4* was also down‐regulated in MM patients from the CoMMpass study who carried *FAM46C* non‐sense mutations. Several genes down‐regulated by *FAM46C* inactivation have been associated with glycosylation. Down‐regulation of these factors might affect glycosylation patterns of PCs, which may have implications for cell signalling or cell‐matrix interactions.^41^ Alternatively, down‐regulation of these genes may respond to the lowered glycosylation demand arising from decreased Ig production, as Igs are glycosylated proteins.^42^ This hypothesis is supported by the fact that some of them (*DDOST, RPN1* and *LMAN1*) are up‐regulated in PCs from mice (MSigDB M1487 geneset), suggesting a greater demand for these factors during PC differentiation to glycosylate Igs.

Inactivation of FAM46C is expected to induce pleiotropic effects in the cells, not only by deregulation of mRNAs, but also because changes in poly(A) tail length may affect mRNAs translatability giving rise to modifications in the proteome. Here, we found that FAM46C inactivation substantially increased the migratory ability of MM cells. We found that some of the few genes deregulated in the *FAM46C* KO clones, such as *MAGED1, RHOBTB1* and *MALAT1*, had previously been associated with cell migration^20^, ^22^, ^23^, ^43^ *MALAT1* was found to be up‐regulated by *FAM46C* inactivation in the three HMCLs used in this study. This lncRNA was overexpressed in a wide variety of solid tumours and also in haematological malignancies, including MM.^43^, ^44^, ^45^ Moreover, high levels of expression of *MALAT1* in MM have been associated with the onset of the disease, progression from normal PCs to MM and extramedullary dissemination.^44^, ^45^, ^46^, ^47^ *MAGED1* was down‐regulated in JJN3 FAM46C KO clones and RHOBTB1 in two (JJN3 and U266) out of the three HMCLs analysed. The down‐regulation of *MAGED1* has also been reported in the XG1 HMCL knocked out for *FAM46C*.^16^ Although the down‐regulation of these factors by FAM46C inactivation might contribute to the great migration ability observed in JJN3 FAM46C KO cells, this effect seems to be cell type‐specific and genetic background‐dependent. Consistent with our findings, a recent study has demonstrated that overexpression of FAM46C in hepatocellular carcinoma reduced cell migration and invasion.^48^ The authors showed that forced expression of the ncPAP suppressed EMT. However, we found here that the increased migratory ability of *FAM46C* KO MM cells seems to be EMT‐independent. Conversely, we found that the increased rate of migration induced by *FAM46C* loss depended on PI3K‐Rac1 activation in the KO cells, which may have important therapeutic implications, as patients with *FAM46C* mutations are likely to benefit from PI3K or Rac1 inhibitors^49^, ^50^ (Figure 7).

Future investigation should focus on the molecular mechanisms that explain the connections between FAM46C inactivation, the lowering of several ER‐resident proteins and the UPR. Moreover, additional studies are needed to correlate all the findings obtained in MM cell lines with the phenotypes observed in patients, especially regarding migration of *FAM46C*‐mutated myeloma cells and its relation with MALAT1 up‐regulation.

## CONFLICT OF INTEREST

The authors declare no conflict interest.

## AUTHOR CONTRIBUTIONS

ABH designed the study, performed most of the experiments and wrote the paper. DQ carried out the siRNA and qRT‐PCR experiments. LAC performed all the bioinformatic and statistical analyses. RGS supervised the statistical analyses and contributed to the research tools. MVM revised the manuscript and contributed to the research tools. NCG supervised the experiments, corrected and approved the final version of the manuscript.

## Data Availability

The data that support the findings of this study are available on request from the corresponding author. Additional methods are detailed in Appendix S1.
