# Supplementary figures and images for: FAM46C controls antibody production by the polyadenylation of immunoglobulin mRNAs and inhibits cell migration in multiple myeloma
Source: J Cell Mol Med. 2020 Mar 6;24(7):4171–82. doi: 10.1111/jcmm.15078 (PMC7171423; doi:10.1111/jcmm.15078)

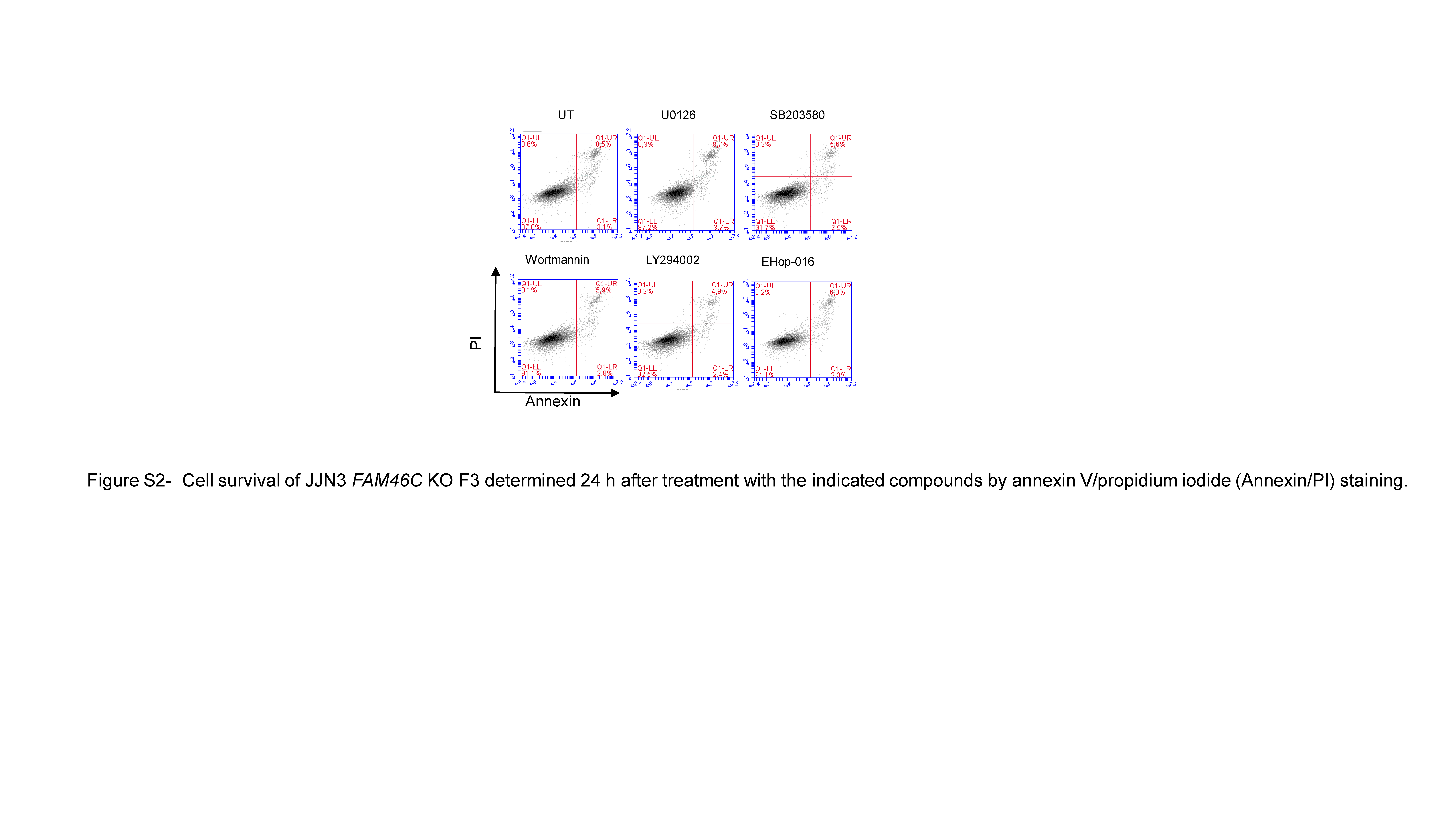

Supplement: Supplementary file 2 [file JCMM-24-4171-s002.tiff]

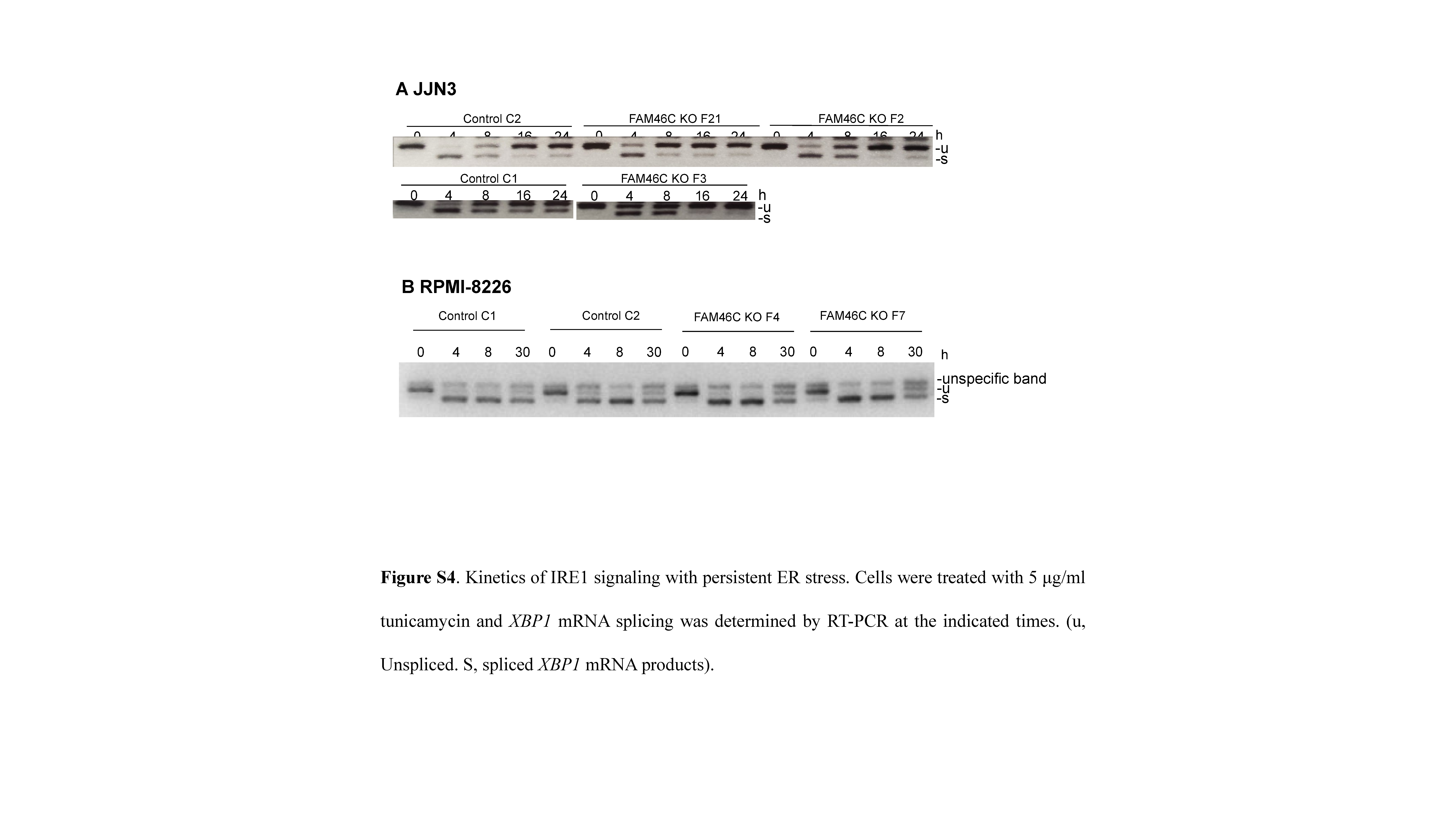

Supplement: Supplementary file 4 [file JCMM-24-4171-s004.tiff]
