# Supplementary material for: FAM46C controls antibody production by the polyadenylation of immunoglobulin mRNAs and inhibits cell migration in multiple myeloma
Source: J Cell Mol Med. 2020 Mar 6;24(7):4171–82. doi: 10.1111/jcmm.15078 (PMC7171423; doi:10.1111/jcmm.15078)
Supplement: Supplementary file 5 [file JCMM-24-4171-s005.docx]

| Primer name | Primer sequence |
| --- | --- |
| \| FAM46C-FOR \|  \| \| --- \| --- \| | 5′-ATGGCAGAGGAGAGCAGCTGTA-3′ |
| FAM46C-REV | 5′-GGTAAGTCTCCAACTTCCTCT-3′ |
| FAM46C-REV2 | 5′-GAAGGGAACACAGAACCACAT-3′ |
| GAPDH-FOR | 5′-GGGTGGAATCATATTGGAACATGTA-3′ |
| GAPDH-REV | 5′-CAGGGCTGCTTTTAACTCTGGTAA-3′ |
| IGKC | 5′-AAGAGCTTCAACAGGGGAGAGTGTTAG-3′ |
| SSR4 | 5′-ATTTCCATCATCCCGCCTCTGTTTACA-3′ |
| IGLC | 5′-ATGAAGGGAGCACCGTGGAGAAGACA-3′ |
| BIP | 5′-GTGTACCCTGGGGCAATAGGGTTAAAT-3′ |
| IGHA1ns | 5′-TTCACACAGAAGACCATCGACCGCTT-3′ |
| IGHA1s | 5′-TCATGGCGGAGGTGGACGGCACCTGCTACT-3′ |
| IGHEns | 5′-AGACCTCCCTCGACTACACCAA-3′ |
| IGHEs | 5′-CTGGCCAGACCTTCTGTCCACTGTTGCAA-3′ |
| XBP1 F | 5′-TTA CGA GAG AAAA CTC ATG GCC-3′ |
| XBP1 R | 5’-GGG TCC AAG TTG TCC AGA ATGC-3’ |

**Table S1.** PCR primers
