# Supplementary material for: FAM46C controls antibody production by the polyadenylation of immunoglobulin mRNAs and inhibits cell migration in multiple myeloma
Source: J Cell Mol Med. 2020 Mar 6;24(7):4171–82. doi: 10.1111/jcmm.15078 (PMC7171423; doi:10.1111/jcmm.15078)
Supplement: Supplementary file 6 [file JCMM-24-4171-s006.docx]

| Probe Set ID | Gene Symbol | F2 vs C1 | F2 vs C2 | F2 vs C5 | F3 vs C1 | F3 vs C2 | F3 vs C5 | F21 vs C1 | F21 vs C2 | F21 vs C5 | Number of Comparisons  FC < 1.5 |
| --- | --- | --- | --- | --- | --- | --- | --- | --- | --- | --- | --- |
| 11715506_a_at | ***MAGED1*** | **-5.74** | **-6.12** | **-5.14** | **-1.74** | **-1.85** | **-1.56** | **-2.20** | **-2.34** | **-1.97** | 9 |
| 11715507_s_at | *MAGED1* | **-6.93** | **-8.12** | **-6.83** | **-1.61** | **-1.88** | **-1.58** | **-2.09** | **-2.45** | **-2.06** | 9 |
| 11715819_a_at | ***LRPAP1*** | **-2.24** | **-2.34** | **-2.24** | **-1.53** | **-1.60** | **-1.53** | **-1.56** | **-1.63** | **-1.56** | 9 |
| 11719551_s_at | ***RHOBTB1*** | **-3.98** | **-1.91** | **-1.91** | **-3.31** | **-1.59** | **-1.59** | **-3.34** | **-1.60** | **-1.60** | 9 |
| 11723293_at | ***GPX7*** | **-2.25** | **-2.17** | **-2.95** | **-1.67** | **-1.61** | **-2.20** | **-1.71** | **-1.65** | **-2.25** | 9 |
| 11726583_s_at | ***CKAP4*** | **-1.90** | **-1.87** | **-1.77** | **-1.74** | **-1.71** | **-1.62** | **-1.68** | **-1.66** | **-1.56** | 9 |
| 11728451_a_at | ***PCOLCE2*** | **-9.35** | **-8.79** | **-2.10** | **-8.39** | **-7.89** | **-1.88** | **-8.48** | **-7.97** | **-1.90** | 9 |
| 11728701_a_at | ***CD55*** | **-2.37** | **-2.31** | **-1.53** | **-3.19** | **-3.11** | **-2.05** | **-2.42** | **-2.36** | **-1.56** | 9 |
| 11729887_at | ***HACD1*** | **-4.26** | **-5.80** | **-4.63** | **-2.09** | **-2.84** | **-2.27** | **-1.99** | **-2.71** | **-2.17** | 9 |
| 11752832_x_at | *CD55* | **-2.28** | **-2.08** | **-1.55** | **-2.61** | **-2.37** | **-1.77** | **-2.83** | **-2.58** | **-1.92** | 9 |
| 11755084_x_at | *CD55* | **-2.20** | **-1.95** | **-1.52** | **-2.54** | **-2.26** | **-1.76** | **-2.49** | **-2.21** | **-1.72** | 9 |
| 11755335_a_at | *C1orf21* | **-2.48** | **-2.30** | **-2.28** | **-4.21** | **-3.90** | **-3.87** | **-1.75** | **-1.62** | **-1.61** | 9 |
| 11756557_x_at | *LRPAP1* | **-2.24** | **-2.40** | **-2.27** | **-1.54** | **-1.65** | **-1.56** | **-1.57** | **-1.68** | **-1.59** | 9 |
| 11756693_a_at | *MAGED1* | **-7.48** | **-6.70** | **-8.06** | **-1.90** | **-1.71** | **-2.05** | **-1.94** | **-1.73** | **-2.09** | 9 |
| 11757585_x_at | *MAGED1* | **-5.67** | **-6.28** | **-5.26** | **-1.70** | **-1.89** | **-1.58** | **-2.23** | **-2.48** | **-2.07** | 9 |

**Table 1.** Genes downregulated in *FAM46C* KO clones (F2, F3, F21) versus WT cells (C1, C2, C5)
