# Supplementary material for: FAM46C controls antibody production by the polyadenylation of immunoglobulin mRNAs and inhibits cell migration in multiple myeloma
Source: J Cell Mol Med. 2020 Mar 6;24(7):4171–82. doi: 10.1111/jcmm.15078 (PMC7171423; doi:10.1111/jcmm.15078)
Supplement: Supplementary file 9 [file JCMM-24-4171-s009.docx]

| Probe Set ID | Gene Symbol | F2 vs C1 | F2 vs C2 | F2 vs C5 | F3 vs C1 | F3 vs C2 | F3 vs C5 | F21 vs C1 | F21 vs C2 | F21 vs C5 | Number of comparisons  FC> 1.5 |
| --- | --- | --- | --- | --- | --- | --- | --- | --- | --- | --- | --- |
| 11730370_a_at | ***EIF4E3*** | **2.29** | **5.79** | **3.37** | 1.16 | **2.94** | **1.71** | **1.86** | **4.71** | **2.74** | 8 |
| 11730369_a_at | *EIF4E3* | **1.76** | **3.62** | **2.15** | 1.07 | **2.20** | 1.30 | **1.83** | **3.76** | **2.24** | 7 |
| 11730371_a_at | *EIF4E3* | **2.61** | **3.72** | **2.72** | 1.22 | **1.73** | 1.27 | **1.96** | **2.79** | **2.04** | 7 |
| 11757694_s_at | *EIF4E3* | **2.16** | **3.53** | **2.48** | 1.07 | **1.75** | 1.23 | **1.82** | **2.97** | **2.09** | 7 |
| 11720044_at | ***SKAP2*** | **1.59** | **2.33** | 1.43 | **1.67** | **2.45** | **1.51** | 1.11 | **1.64** | 1.01 | 6 |
| 11723112_a_at | ***CCDC84*** | **1.82** | **1.57** | 1.40 | **1.78** | **1.54** | 1.37 | **1.76** | **1.53** | 1.36 | 6 |
| 11730716_a_at | ***PAGE5*** | **9.67** | **9.90** | **9.31** | **120.45** | **123.37** | **115.94** | 1.22 | 1.25 | 1.17 | 6 |
| 11730717_x_at | *PAGE5* | **9.57** | **10.01** | **9.70** | **84.47** | **88.36** | **85.64** | 1.31 | 1.37 | 1.33 | 6 |
| 11732808_a_at | ***PROK2*** | **2.18** | **2.14** | **2.41** | 1.26 | 1.23 | 1.39 | **2.81** | **2.76** | **3.11** | 6 |
| 11732809_a_at | *PROK2* | **1.95** | **2.15** | **2.19** | 1.28 | 1.42 | 1.44 | **2.30** | **2.54** | **2.59** | 6 |
| 11733415_a_at | *PAGE5* | **23.99** | **27.25** | **22.86** | **272.07** | **308.99** | **259.19** | 1.13 | 1.28 | 1.07 | 6 |
| 11735570_a_at | ***SORBS2*** | **6.58** | **6.81** | **6.72** | 1.09 | 1.13 | 1.11 | **5.55** | **5.75** | **5.67** | 6 |
| 11745286_a_at | *EIF4E3* | **2.06** | **2.58** | **2.19** | 1.14 | 1.42 | 1.20 | **1.68** | **2.10** | **1.78** | 6 |
| 11745723_a_at | ***MALAT1*** | **1.70** | 1.21 | **1.50** | **3.99** | **2.84** | **3.53** | **1.68** | 1.19 | 1.48 | 6 |
| 11755605_s_at | *MALAT1* | **1.89** | **5.59** | 1.49 | 1.46 | **4.31** | 1.15 | **2.08** | **6.15** | **1.64** | 6 |
| 11757872_s_at | ***PLSCR1*** | **1.81** | **2.05** | 1.31 | **1.83** | **2.06** | 1.32 | **1.63** | **1.84** | 1.18 | 6 |
| 11762748_s_at | ***TRA2A*** | 1.21 | **1.59** | 1.45 | **2.03** | **2.68** | **2.44** | 1.27 | **1.68** | **1.53** | 6 |

**Table 2.** Genes upregulated in *FAM46C* KO clones (F2, F3, F21) versus WT cells (C1, C2, C5)
