# Supplementary material for: FAM46C controls antibody production by the polyadenylation of immunoglobulin mRNAs and inhibits cell migration in multiple myeloma
Source: J Cell Mol Med. 2020 Mar 6;24(7):4171–82. doi: 10.1111/jcmm.15078 (PMC7171423; doi:10.1111/jcmm.15078)
Supplement: Supplementary file 14 [file JCMM-24-4171-s014.docx]

**Supplementary methods**

**RT-PCR analysis of *XBP1* mRNA splicing**

RNA was reverse-transcribed to cDNA and used as a template for PCR amplification. DNA corresponding to unspliced or spliced forms of *XBP1* mRNA was generated with the following primers: XBP1 F and XBP1 R. PCR conditions were: 94°C for 5 min; 94°C for 1 min; 58°C for 30 s; 72°C for 30 s; 72°C for 5 min with 35 cycles of amplification. PCR products were resolved on a 2.5% agarose/1x TAE gel.

**Western blot**

Cells were washed with PBS and lysed in RIPA buffer containing protease inhibitors (Complete, Roche Applied Science, Indianapolis, IN, USA) and phosphatase inhibitors (PhosSTOP™, Roche). Protein concentration was measured using the Bradford assay (BioRad). Protein samples (25 μg/lane) were subjected to SDS-PAGE and transferred to PVDF membrane (BioRad). Anti-FAM46C antibodies were purchased from Proteintech (Manchester, UK), Anti-MAGED1, anti-MMP2 and anti-MMP-9 antibodies were obtained from Santa Cruz Biotechnology. Anti-RHOBTB1, anti-PROK2 and anti-TRA2 were from Abcam (Cambridge, UK). Anti-N-cadherin, anti-Twist, anti-Slug, anti-IRF4, anti-ERK, anti-BCL2, anti-Ig Kappa and anti-Ig lambda were purchased from Santa Cruz Biotechnology and anti-p-ERK2 was obtained from Cell Signaling (Beverly, MA, USA). Horseradish peroxidase-linked anti-rabbit (Abcam), anti-mouse (Abcam) or anti-goat antibodies (Santa Cruz Biotechnology) were used as secondary antibodies at 1:10.000 dilution. Anti-β-actin (Sigma-Aldrich, St. Louis, MO, USA) was used as an internal control for protein loading. Immunoblots were incubated for 1 h at RT and developed using enhanced chemiluminescence western blotting detection reagents (BioRad).

**Reagents**

U0126, SB203580, wortmannin, LY294002, EHop-016 and tunicamycin were purchased from MedChemExpress (Sollentuna, Sweden). Melphalan and dexamethasone were acquired from Sigma-Aldrich and bortezomib from LC Laboratories (Woburn, MA, USA).

**Statistical analysis**

The statistical significance of group differences was assessed using Student's unpaired two-tailed *t*-test, unless otherwise specified. Data are summarized as the mean and standard deviation (SD) of at least three determinations.
